# A novel X-ray diffraction approach to assess the crystallinity of regenerated cellulose fibers

**DOI:** 10.1107/S205225252200570X

**Published:** 2022-06-11

**Authors:** Luigi Gentile, Herbert Sixta, Cinzia Giannini, Ulf Olsson

**Affiliations:** a Department of Chemistry, Bari Università, Via Edoardo Orabona, Bari, Italy; b Bari unit, Consorzio Interuniversitario Per Lo Sviluppo Dei Sistemi A Grande Interfase, Via della Lastruccia 3, Sesto Fiorentino 50019, Italy; c Department of Bioproducts and Biosystems, School of Chemical Engineering, Aalto University, PO Box 16300, Helsinki 00076, Finland; d Institute of Crystallography - National Research Council, Via Amendola 122/O, Bari 70126, Italy; e Physical Chemistry, Lund University, PO Box 124, Lund 22100, Sweden

**Keywords:** cellulose, crystallinity, wide-angle X-ray scattering, fibers, framework-structured solids and amorphous materials, nanostructure, crystalline domains, X-ray diffraction, orientations, draw ratios

## Abstract

Here, a new accurate approach is presented to quantify the degree of crystallinity of regenerated cellulose textile fibers using wide-angle X-ray scattering. The approach is based on the observation that the contributions to the scattering from crystalline and amorphous domains of the fibers can be separated due to their different degree of orientation with respect to the fiber direction. The method is tested on Ioncell-F fibers, dry jet wet spun with different draw ratios from an ionic liquid solution. The analysis output includes, apart from an accurate estimate of the fiber crystallinity, the degrees of orientation of the cellulose nanocrystals and the cellulose chains in the amorphous domains.

## Abstract 2

By using wide-angle X-ray scattering, contributions from amorphous and crystalline domains are detected separately in cellulose fibers, which enables an accurate determination of regenerated cellulose-fiber crystallinity.

## 1. Introduction

Cellulose is the most abundant biopolymer on Earth, whereas wood-based cellulose represents a particularly important and renewable raw material, for example for textile fibers (Woodings, 2001 ▸; Sixta *et al.*, 2015 ▸). It is a crystalline polymer with such a high melting point that it cannot be melted without chemical decomposition. Hence, shaping cellulose materials generally requires dissolution and precipitation (regeneration). However, because of its highly stable crystalline state, cellulose is also fascinatingly insoluble in simple solvents, over the whole polarity scale from polar to non-polar (Medronho *et al.*, 2012 ▸). It is partly soluble in strong alkali solutions (Budtova & Navard, 2016 ▸; Gentile & Olsson, 2016 ▸; Gubitosi *et al.*, 2016 ▸) and in certain ionic liquids (Idström *et al.*, 2017 ▸; Zhang & Wang, 2017 ▸), which are the basis of the viscose and lyocell fiber processes, respectively (Luo *et al.*, 2001 ▸). These regenerated cellulose fibers are spun from the solutions and coagulated in an anti-solvent, involving the recrystallization of cellulose, into the crystalline allomorph generally referred to as cellulose II (Langan *et al.*, 2001 ▸), differing from the native allomorph cellulose I (Langan *et al.*, 2005 ▸). As is common for crystalline polymers, crystallization is only partial and there is, in the end, a mixture of crystalline and amorphous domains (Rosa & Auriemma, 2013 ▸).

Fiber structural characteristics, such as crystallinity, crystal orientation and anisotropy of amorphous domains, that can be partly tuned by process parameters (Asaadi *et al.*, 2018 ▸), are considered to significantly affect their mechanical properties (Sharma *et al.*, 2019 ▸). By crystallinity we here mean the overall fraction, from zero to one, of the material that is crystalline, as opposed to amorphous. Detailed structural information of materials is often and conveniently obtained using X-rays (Giannini *et al.*, 2020 ▸). X-ray diffraction (XRD), in fact, is a very commonly used technique used to evaluate, in particular, the crystallinity of regenerated cellulose fibers. A few different XRD-based approaches for assessing the crystallinity have been proposed over the years. Discussions of these, and comparisons, can be found in several reviews (Lindner *et al.*, 2015 ▸; French, 2020 ▸; Ahvenainen *et al.*, 2016 ▸). Most of these methods involve the analysis of the 1D radially averaged powder diffractogram. Evaluating crystallinity from XRD data implies quantifying the individual contributions to the overall scattering pattern from the crystalline and amorphous domains. A major drawback of using the 1D powder diffractogram in this respect is that the scattering from the amorphous domains is hidden within the crystal diffractogram background. Driemeier *et al.* (Driemeier & Calligaris, 2011 ▸; Oliveira & Driemeier, 2013 ▸) have extended the Rietveld refinement method to analyze 2D data, including the case of a preferred crystal orientation. However, this approach still does not explicitly identify the scattering from amorphous domains. Quantitative comparisons between the most common methods, including 2D Rietveld refinement, were recently presented by Ahvenainen *et al.* (2016 ▸).

To overcome this issue, we are here validating a new methodology to obtain a more rigorous crystallinity determination on the basis of the analysis of wide-angle X-ray fiber diffraction profiles from oriented fibers, collected with a 2D detector. The method is applicable to fibers having a sufficient degree of anisotropy, therefore displaying the typical fiber cross-diffraction pattern, with a significantly different degree of crystal and amorphous chain orientations. Following a recent publication by Gubitosi *et al.* (2021 ▸), the method involves analyzing the azimuthal angular dependence of the recorded intensity within a given *q* range, with *q* being the magnitude of the scattering vector. Crystalline and amorphous contributions can indeed be distinguished in selected *q* ranges by diffraction spots with different degrees of orientation (different intensity distribution along the azimuth). The present work involves a new and extended analysis of the 2D fiber diffraction data recorded by Asaadi *et al.* (2018 ▸) from Ioncell-F fibers, dry jet wet spun with different draw ratios, DR, from an ionic liquid solution. The draw ratio is here defined as the ratio between pick-up velocity and extrusion velocity in the spinning process. We will first describe how crystal and amorphous chain orientations be separately assessed. After that we address the crystallinity, by individually integrating the scattered intensities, selected in *q* space, as coming from crystalline and amorphous domains. Finally, the article ends with some concluding remarks.

## 2. Crystal and amorphous chain orientation

**Figure 1:**
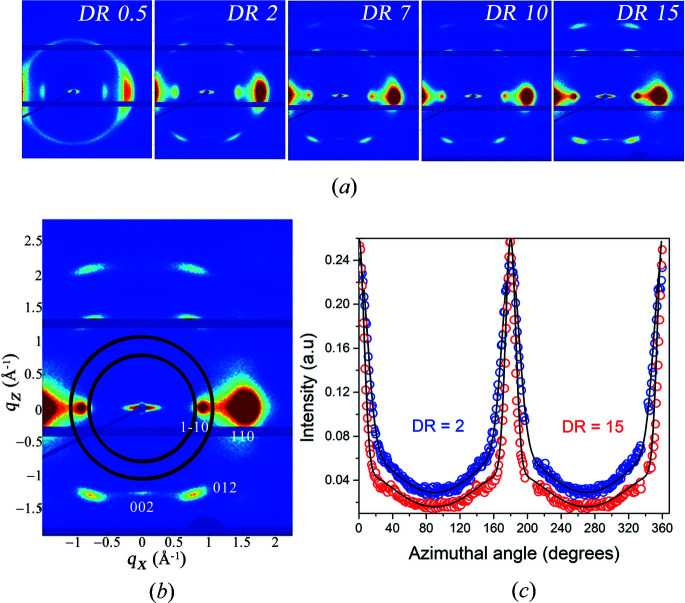
(*a*) From left to right, 2D fiber diffraction patterns obtained at different draw ratios DR = 0.5, 2, 7, 10 and 15. (*b*) A 2D diffraction pattern from the DR = 15 fibers, with assignment of diffraction spots according to cellulose II. The space between the two circles indicates the *q* band of 0.80–0.95 Å^−1^, which includes the 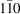 reflection. (*c*) Azimuthal plots of the intensity in the *q* band of 0.80–0.95 Å^−1^, for two different draw ratios, DR = 2 (blue open circles) and DR = 15 (red open circles). The solid black lines are model fits corresponding to a linear combination of two Gaussian functions, see equation (1).

Fibers of different draw ratios (DR = 0.5–15) were investigated by fiber XRD. In Fig. 1 ▸(*a*), we present a few selected 2D diffraction patterns. As can be seen, the diffraction patterns are highly anisotropic, with individual diffraction spots, showing that the crystalline domains in these fibers are highly oriented. A partial peak indexing, according to the allomorph cellulose II, is shown in Fig. 1 ▸(*b*). Comparing the 2D patterns, we can see qualitatively that the diffraction spots become narrower and sharper, along the azimuth, as the draw ratio increases. For a more quantitative picture, we have analyzed how the intensity varies with the azimuthal angle, φ, within a suitable *q* band, 0.80–0.95 Å^−1^, illustrated in Fig. 1 ▸(*b*). As was shown by Gubitosi *et al.* (2021 ▸), it is possible to determine both the orientation distribution of crystallites as well as the orientation distribution of the cellulose chains in the amorphous domains, from an azimuthal plot. Azimuthal plots, *I*(φ), for two fibers, DR = 2 and 15, are presented in Fig. 1 ▸(*c*). The data are well described by a superposition of two Gaussian functions, one narrow and one broad, with peaks at 0, 180 and 360° (same as 0°), where we identify the narrow component as resulting from the crystalline domains and the broad component as resulting from the amorphous domains. For quantitative analysis, we fit the data with a model function, *I*(φ), that we write as

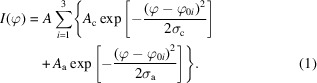

**Figure 2:**
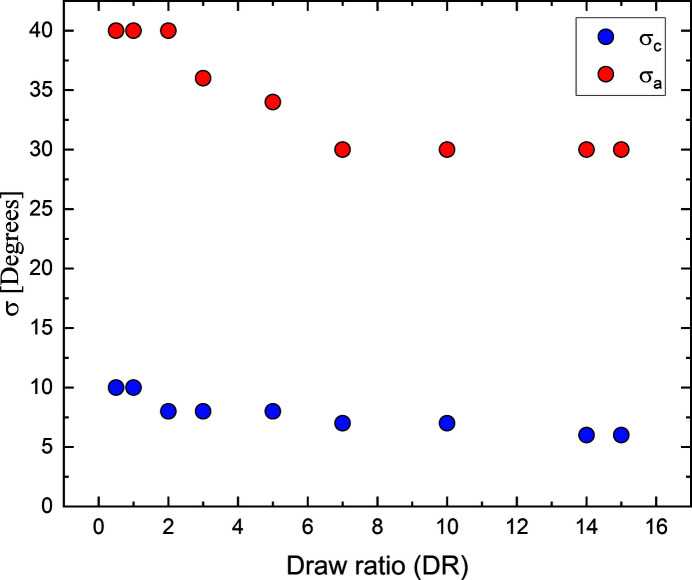
Degree of orientation, expressed as the standard deviation of a Gaussian distribution, of crystalline domains (σ_c_, blue filled circles) and cellulose chains in the amorphous part of the fibers (σ_a_, red filled circles), plotted as a function of the draw ratio.

Here, *A* is the overall signal amplitude, and *A*_c_ and *A*_a_ are the relative amplitudes of the crystalline and amorphous contributions, respectively, with *A*_c_ + *A*_a_ = 1. The sum runs over the three different peaks located at φ = 0, 180 and 360°, and σ_c_ and σ_a_ are the standard deviations of the crystalline and amorphous contributions, respectively. However, note that 0 and 360° correspond to the same peak. The best fits of equation (1) to the data are shown as solid lines in Fig. 2 ▸. As can be seen, the model describes the data well. For the high draw ratio (DR = 15) we obtain σ_c_ = 6° and σ_a_ = 30°, while for the lower draw ratio (DR = 2), we find slightly lower degrees of orientation, σ_c_ = 10° and σ_a_ = 40°. The fact that the azimuthal profile can be described by a linear combination of two discrete Gaussian functions implies that we can describe the fibers as two-phase systems (crystalline + amorphous).

The same azimuthal analysis was also performed on other draw ratios, and the variations of σ_c_ and σ_a_ with draw ratio are summarized in Fig. 2 ▸. As can be seen, the degree of crystal orientation, quantified as the standard deviation σ_c_, increases with increasing draw ratio. However, the increase is relatively low because the degree of orientation is already high for DR = 0.5. For the amorphous chains, σ_a_ decreases from 40 to 30° when draw ratio increases from 0.5 to 7. For DR > 7 there is essentially no further change.

## 3. Fiber crystallinity

As shown in the previous section, we are able to identify separately the individual contributions to the wide-angle X-ray scattering (WAXS) pattern coming from the amorphous and crystalline domains [Fig. 2 ▸(*c*)]. This allows for determining the degree of fiber crystallinity, *i.e.* the volume fraction of the crystalline domains, ϕ_c_, from the ratio (de Jeu, 2016 ▸):

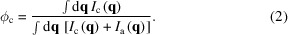

**Figure 3:**
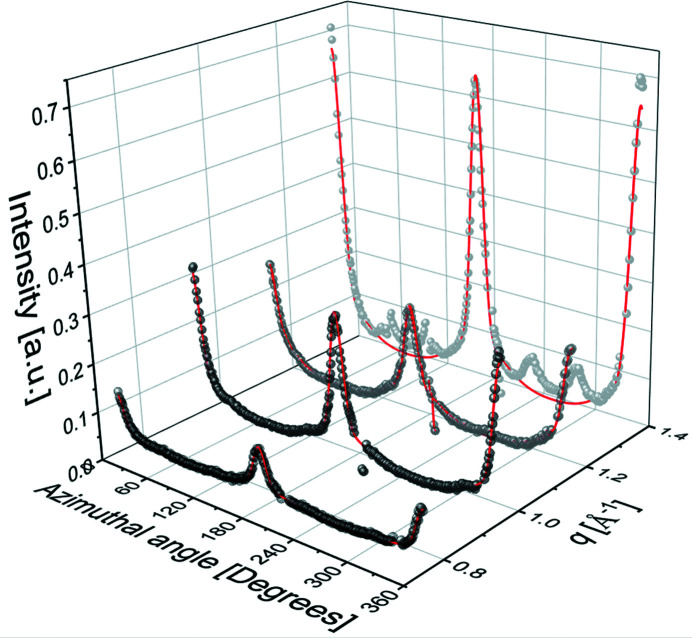
Azimuthal plots of four selected *q* bands: 0.7–0.8, 0.9–1.0, 1.1–1.2 and 1.3–1.4 Å^−1^. The data are shown with circles. The red solid lines represent fits of equation (1) to the data. For the *q* band of 1.3–1.4 Å^−1^, the small peaks corresponding to the 012 reflections are neglected in the fit.

Here, *I*_c_(**q**) is the intensity diffracted from the crystalline domains and *I*_a_(**q**) is the intensity scattered from the amorphous domains. Azimuthal plots of individual *q* bands of width 0.1 Å^−1^ in the *q* range of 0.6–1.9 Å^−1^ were fitted using equation (1) to obtain the two relative amplitudes, *A*_c_ and *A*_a_ (*A*_c_ + *A*_a_ = 1), and the total amplitude, *A*, together with the two standard deviations, σ_c_ and σ_a_. As an illustration of the approach, in Fig. 3 ▸ we have plotted the azimuthal intensities of four selected *q* bands, together with the corresponding fits, using equation (1). Within the *q* band of 1.3–1.4 Å^−1^ there are four additional small peaks observed, corresponding to the 012 reflections [Fig. 1 ▸(*b*)]. These peaks are neglected in the fits of the main equatorial peaks as their contribution to the total integral is only minor.

**Figure 4:**
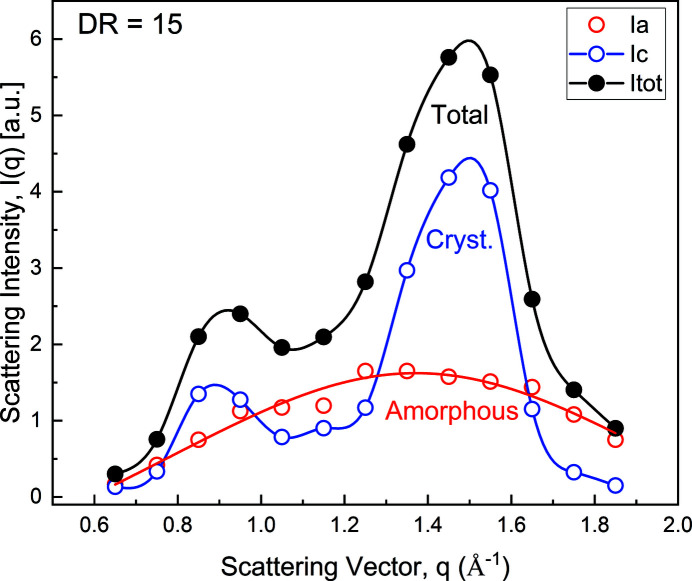
Individual contributions to the total WAXS pattern from amorphous and crystalline domains, where DR = 15. Solid lines are merely guides to the eye. However, for the amorphous domains, a Lorentzian fit is reported.

Within each *q* band, the integrated crystalline and amorphous scattered intensities were evaluated as *I*_c*i*_ = *A*_*i*_σ_c_ *A*_c_ and *I*_a*i*_ = *A*_*i*_σ_a_ *A*_a_, respectively, where the index *i* denotes the different *q* bands. Then, ϕ_c_ was calculated by summing up the contributions from the 13 individual *q* bands, 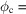 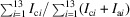 . Within this 0.1 Å^−1^ *q*-resolution, we are also able to reconstruct the overall integrated amorphous and crystalline intensities, compared with the total intensity, as illustrated in Fig. 4 ▸ for the case of DR = 15. As can be seen, the amorphous contribution is significant but is completely hidden in the total scattering pattern. The solid lines in Fig. 4 ▸ are merely guides to the eye. For the amorphous contribution the solid line corresponds to a Lorentzian fit to the data, *I*(*q*) = *A*[1 + ξ^2^(*q* − *q*_0_)^2^]^−1^, where *q*_0_ = 1.37 Å^−1^ is the peak position, reporting on nearest neighbor separation, *A* = 9.1 is the amplitude and ξ = 2.1 Å^−1^ can be viewed as a correlation length of nearest neighbor separation fluctuation. This figure clearly demonstrates that assessing accurately the fiber crystallinity from WAXS data requires explicit access to the individual amorphous and crystalline contributions.

**Figure 5:**
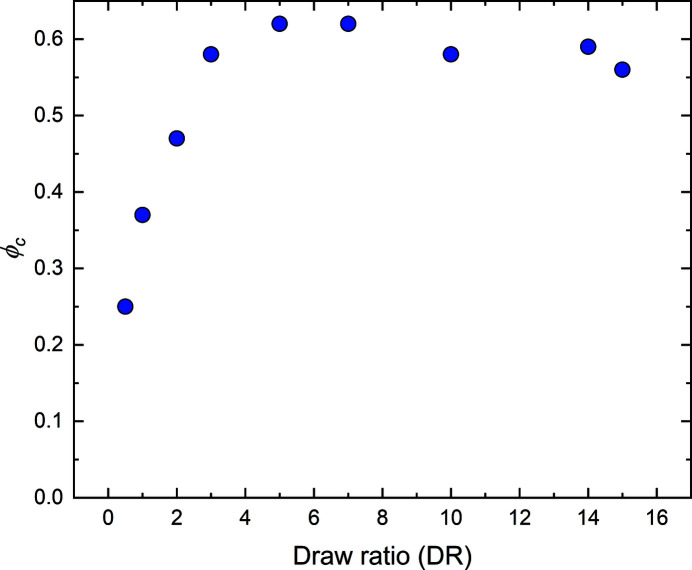
Fiber crystallinity, *i.e.* the volume fraction of crystalline domains, ϕ_c_, obtained at different draw ratios.

In Fig. 5 ▸ we have plotted the evaluated ϕ_c_ for the different draw ratios investigated here. As can be seen, ϕ_c_ increases strongly with increasing draw ratio for lower draw-ratio values, and essentially levels off at ϕ_c_ = 0.6 ± 0.05 for DR ≥ 5. Drawing the fibers is known to increase the fiber crystallinity (Grosberg & Khokhlov, 2010 ▸). Drawing increases the degree of orientation of the individual cellulose molecules, which facilitates the formation of crystals. Furthermore, the leveling off of ϕ_c_ at higher draw ratio correlates with the leveling off of σ_a_ at a value of 30° for DR ≥ 7 (Fig. 3 ▸). These observations also correlate with how the mechanical properties of the fibers vary with draw ratio (Asaadi *et al.*, 2018 ▸; Sixta *et al.*, 2015 ▸). The tensile strength increases by approximately a factor of 2 when draw ratio increases from 0.5 to 7, while in the draw-ratio range of 7–15 only a minor increase of the tensile strength was observed, with no significant difference in the draw-ratio range of 10–15. Also, the full stress–strain curve does not change significantly between DR = 7 and DR = 15 (Sixta *et al.*, 2015 ▸).

## 4. Conclusions

We have shown that it is possible to quantitatively determine the relative contributions from amorphous and crystalline domains in an X-ray diffraction pattern from regenerated cellulose fibers. A requirement is that the different contributions show significantly different degrees of orientation within the fiber, allowing them to be separated when analysing the dependence of the scattered intensity on the azimuthal angle. Identifying the crystalline and amorphous contributions separately allows not only for determining their individual degree of orientation, but also for a more accurate determination of fiber crystallinity, as it involves the measurement of both contributions. The proposed approach should represent an improvement compared with crystallinity assessments based on 1D powder pattern profiles, where the contributions from amorphous domains, in general, are hidden in the background. We find that the azimuthal profiles from the present fibers are accurately described by a sum of two Gaussian contributions that we identify with the crystalline and amorphous contributions. This observation is consistent with a two phase (crystalline + amorphous) description of the material.
